# Nanophotonic-assisted precision enhancement of weak measurement using spin Hall effect of light

**DOI:** 10.1515/nanoph-2022-0447

**Published:** 2022-09-20

**Authors:** Minkyung Kim, Dasol Lee, Yeseul Kim, Junsuk Rho

**Affiliations:** Department of Mechanical Engineering, Pohang University of Science and Technology (POSTECH), Pohang 37673, Republic of Korea; School of Mechanical Engineering, Gwangju Institute of Science and Technology (GIST), Gwangju 61005, Republic of Korea; Department of Biomedical Engineering, Yonsei University, Wonju 26493, Republic of Korea; Department of Chemical Engineering, Pohang University of Science and Technology (POSTECH), Pohang 37673, Republic of Korea; POSCO-POSTECH-RIST Convergence Research Center for Flat Optics and Metaphotonics, Pohang 37673, Republic of Korea

**Keywords:** optical spin Hall effect, photonic spin Hall effect, precision, spin Hall shift, weak measurement

## Abstract

The spin Hall effect of light, i.e., the microscopic and spin-dependent transverse splitting of linearly polarized light into circular polarizations at an optical interface, has been considered as a promising candidate for high-precision measurement when combined with a weak measurement technique. However, in those previous demonstrations, the precision is determined by the interface of interest, hindering its versatility. Here, by leveraging the direct correlation of precision with the spin Hall shift, we propose nanophotonic-assisted approaches to increase the precision of the weak measurement by controlling the spin Hall effect of light at the target interface. The refractive index sensing of an isotropic medium is demonstrated as a proof of concept, in which the precision can be increased, in principle, to infinity by placing an index-below-unity slab in the vicinity of the target interface. Furthermore, a single-layer metasurface comprising two-dimensional subwavelength patterns is introduced as an experimentally favorable platform. This study lays the foundation for nondestructive and high-precision investigation of unknown parameters of interfaces and will find wide sensing applications in material science, medical engineering, and other interdisciplinary fields.

## 1 Introduction

**Figure 1:**
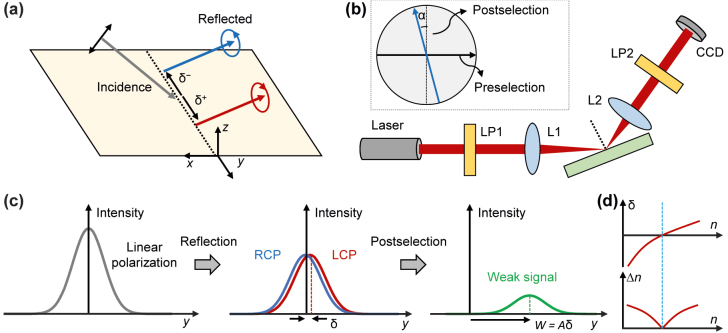
Spin Hall effect of light (SHEL) and its implementation for high-precision measurement. (a) Schematic of the SHEL. (b) Weak measurement setup. Inset: polarizations of preselection and postselection states. (c) Beam intensity profiles of the incident, reflected, and postselected beams along the transverse axis. LP, linear polarization; L, lens; CCD, charge-coupled device. (d) Illustration of *δ* and Δ*n* when *δ* crosses zero.

Recently, advances in nanotechnology have led to intensive studies on nanophotonics. Subwavelength-scale structures designed to exhibit extraordinary optical properties and their interactions with light have enabled several unprecedented phenomena and applications, such as super-resolution imaging [1, 2], virtual and augmented reality [3–5], and powerless cooling technology [6–8]. An interesting area of study in nanophotonics is the spin Hall effect of light [9–15] (SHEL), the photonic analog of the spin Hall effect—transverse spin accumulation of electric current [16, 17]. The SHEL manifests as the spatial transverse splitting of linearly polarized light into two opposite circular polarizations, i.e., left and right circular polarizations (LCP and RCP, respectively), at a planar optical interface (Figure 1a). Whereas the SHEL at natural interfaces is generally much smaller than the wavelength and has been neglected, studies on nanostructured media such as metamaterials and metasurfaces have demonstrated that it can be significantly amplified [18–34].

Despite recent efforts, nanophotonics have yet to be implemented to improve the precision of the SHEL-based measurement methods. One of the most promising applications of SHEL is high-precision measurement, in which the spin Hall shift is used as a measurement pointer. A key advantage is that the spin Hall shift responds sensitively to changes in the properties of an interface and can be measured precisely using weak measurement techniques [10] (Figure 1b). Thus, the SHEL has been leveraged to identify the unknown parameters/variables of an interface of interest such as refractive index, concentration, chemical reaction rate, and humidity with high precision [35–44]. Likewise, previous studies have been mainly focused on applying the SHEL-based high-precision measurement scheme to various interfaces and on expanding the measurable quantities. However, in those previous demonstrations, the smallest difference of the spin Hall shift we can experimentally detect depends on the interface of interest and cannot be changed or tuned arbitrarily. Therefore, the precision of these measurements is generally determined by the target interface. This prevents us from investigating certain interfaces, at which the spin Hall shift is large or its derivative with respect to the target parameter is negligible, and compromises its practicality. Therefore, a method to increase the precision of this SHEL-based measurement is in high demand.

In this article, we propose a nanophotonic-assisted approach to substantially enhance the precision of weak measurements using SHEL for the first time. We first demonstrate that the resolution of the measurement, i.e., the smallest difference we can detect through the measurement, is directly proportional to the spin Hall shift and show that the precision can be, in principle, infinite if the spin Hall shift becomes zero by placing an additional nanophotonic structure near the target interface. As a proof of concept, the refractive index sensing of an isotropic lossless medium is presented using an isotropic slab with an index less than unity. Placing the index-below-unity slab in the vicinity of the target interface induces multiple reflections and can make the spin Hall shift cross zero to be favorable for the high precision. In addition, a metasurface composed of a single-layer of dielectric rods is suggested as an experimentally realizable platform. Our work proves that positioning an additional slab or nanopatternings near the target interface to alter the spin Hall shift can enhance precision by several orders of magnitude. This improved precision will enable us to examine unknown parameters of interfaces in a nondestructive manner with extremely high precision that can be extended to a broad range of practical sensing applications.

## 2 Results and discussion

### 2.1 Principle

The spin Hall shift *δ*, which is typically too weak to be directly captured by a detector, can be amplified by several orders of magnitude using the concept of weak measurement [45, 46] (Figure 1b). Two distinct mechanisms contribute to amplification [47]: (i) propagation mediated by two lenses (L1 and L2 in Figure 1b) and (ii) postselection. The first mechanism amplifies the shift by the propagation factor *F* = *z*_*r*_/*z*_*R*_, where *z*_*r*_ is the propagation distance determined by the focal length of the second lens, *z*_*R*_ = *k*_0_*w*^2^/2 is the Rayleigh length, *k*_0_ is the wave vector, and *w* is the beam waist. The second mechanism, which is the central principle of the weak measurement, provides amplification by cot *α*, where *α* is the angular difference between the postselection polarizer and the orthogonal axis of the preselection polarizer (Figure 1b, inset). For instance, the Jones vector of the postselection state is chosen as (−sin *α*, cos *α*) for horizontal preselection (1, 0) and as (cos *α*, sin *α*) for vertical preselection (0, 1). To maximize amplification, *α* is generally small (*α* < 5°). Although filtering the reflected beam with spatially nonuniform polarization through a nearly orthogonal state via postselection weakens the signal strength, it simultaneously amplifies the beam displacement. To sum up, via weak measurement, we obtain a new quantity called a weak signal, displacement of which is significantly increased by the amplification factor *A* = *F* cot *α* (Figure 1c). This amplified shift, often known as the weak value, *W* = *Aδ*, can be several orders of magnitude larger than the original shift by setting the measurement setup parameters properly. This weak value refers to a quantity that corresponds to the shift of the measurement pointer when the measurement accompanies preselection and postselection [45]. The weak value formula can be derived using classical optics [47] and is also summarized in detail in Supplementary Information, Section S1.

Given that the resolution of *W*, i.e., Δ*W*, is a finite constant determined by the pixel size of the detector and/or by irremovable noise, the resolution of *δ* can be significantly reduced by *A* as Δ*δ* = Δ*W*/*A*. This enables high-precision measurement of unknown parameters at the target interface. However, this linear relationship between *W* and *δ* only holds in a weak interaction regime [45, 48], in which

(1) $$\frac{\left|\delta \right|}{w}\ll \min\left(\tan\alpha ,\;\cot\alpha \right).$$

is satisfied and the higher-order weak values are negligible. This equation implies that the SHEL-based high-precision measurement is only available in the weak-coupling regime or when the postselection state deviates sufficiently from the orthogonality of the preselection state, i.e., *α* cannot be indefinitely small. This lower bound of *α* precludes an arbitrarily large *A* and makes the best achievable precision bounded [49]. This is a common limitation that appears in most previous demonstrations of the SHEL-based precision measurements. A modified weak measurement offers the perfect way to measure *δ* even when Eq. (1) is violated, but the amplification factor in the strong-coupling regime converges to zero, thereby limiting the precision [48].

To search for the best precision, we first quantitatively examine the relationship between the resolution and *δ*. Without loss of generosity, we consider refractive index sensing using the SHEL. Note that the measurement target does not have to be the index but can be any other parameter that can change the reflection coefficients, such as conductivity, geometric dimensions, and concentration of solution. Because Eq. (1) can be expressed as *δ* = *ϵw* tan *α* for a small constant *ϵ* when *α* is small (<45°) , the smallest perceivable index difference can be written as

(2) $$\Delta n=\left|\delta {\left(\frac{d\delta }{dn}\;\right)}^{-1}\frac{\Delta W}{\epsilon Fw}\right|.$$

See Supplementary Information, Section S2 for the derivation. Previously, setup parameters, such as the focal lengths of the lenses or *α*, have been deliberately selected to achieve the highest possible precision [49]. However, *δ* has, so far, not been considered as a controllable parameter. In contrast, Eq. (2) demonstrates explicitly that while controlling other parameters cannot make Δ*n* converge to zero, $\delta {\left(\frac{d\delta }{dn}\right)}^{-1}\to 0$ , in principle, results in Δ*n* = 0 and infinite precision (Figure 1d). The zero spin Hall shift satisfies the weak-coupling condition (Eq. (1)) for any nonzero *α* and, therefore, allows *α* to be indefinitely small. In short, Eq. (2) indicates that the infinite precision is achievable by directly controlling *δ*. Note that this infinite precision is unphysical and cannot be accomplished in reality, because the strength of the weak signal approaches zero as *α* → 0. Therefore, in real measurements, the minimum available value of *α* is determined by the minimum detectable signal strength and signal-to-noise ratio. Nevertheless, our approach paves the way toward the significant enhancement of precision, the bound of which is only determined by the signal strength, not by the lower bound of *α*.

To summarize, the fundamental concept of our approach is the modification of the light–matter interaction in nanoscale so that the SHEL does not occur. To this end, another medium is placed in the vicinity of the target interface and induces multiple reflections to change *δ* so that it crosses zero smoothly as illustrated in Figure 1d. In this smoothly crossing regime, the postselection angle *α* can be arbitrarily large, according to Eq. (2), which provides a path to achieve significantly improved precision.

### 2.2 Precision enhancement using an index-below-unity slab

**Figure 2:**
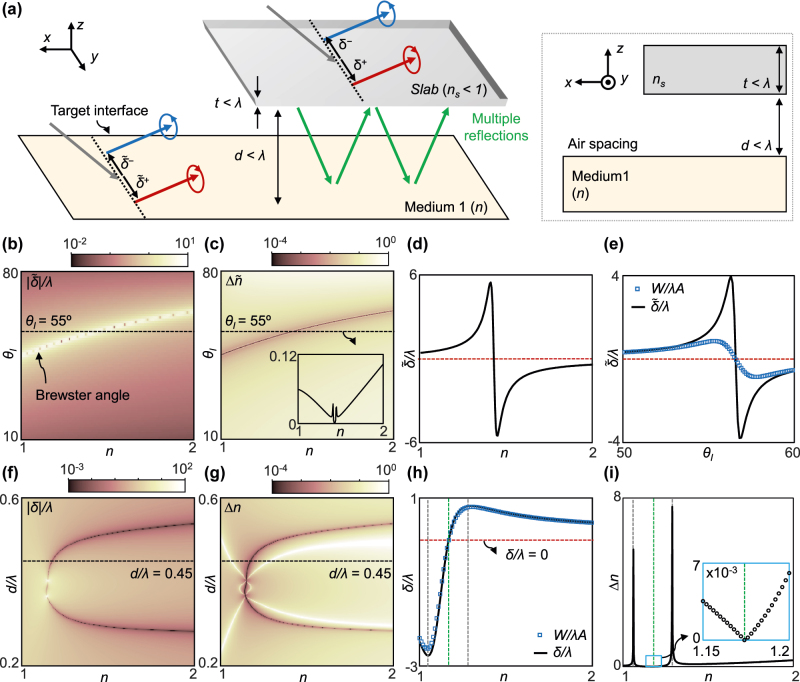
Precision enhancement using an index-below-unity slab. (a) Schematic of reflection in an unknown medium (medium 1) and multiple reflections when a slab with an index of *n*_*s*_ is added. Inset: same schematic in the *z*–*x* plane. (b) Magnitude of the spin Hall shift at the air-medium 1 interface and (c) corresponding index resolution. (d, e) Cross section of the spin Hall shift when (d) *θ*_*I*_ = 55° and (e) *n* = 1.515. (f) Magnitude of the spin Hall shift of the multiple-reflected beam and (g) corresponding index resolution at *θ*_*I*_ = 25°. (h) Spin Hall shift and (i) index resolution when *d*/*λ* = 0.45. All color maps are in the logarithmic scale. The blue markers in (e) and (h) represent the spin Hall shifts obtained by dividing the simulated *W* by *A*. The green and gray lines denote *n* where *δ* = 0 and $\frac{d\delta }{dn}=0$ , respectively.

This section numerically demonstrates the precision enhancement of index sensing achieved using an index-below-unity slab. As a proof of concept, we consider an isotropic lossless medium with an unknown index *n* as a measurement target (Figure 2a). This index of this medium 1 can be identified by measuring the spin Hall shift of the reflected beam via a weak measurement. The spin Hall shifts at an interface characterized by the reflection coefficients of *r*_*s*_ and *r*_*p*_ are known as [50].

(3) $$\begin{array}{c} \delta_{H}^{\pm }/\lambda =\mp \frac{\cot\theta_{I}}{2\pi }\text{Re}\left[\frac{r_{p}\left(r_{p}+r_{s}\right)}{r_{p}^{2}+{\left(\frac{r_{p}+r_{s}}{k_{0}w}cot\theta_{I}\right)}^{2}+{\left(\frac{{\dot{r}}_{p}}{k_{0}w}\right)}^{2}}\right],\\ \delta_{V}^{\pm }/\lambda =\mp \frac{\cot\theta_{I}}{2\pi }\text{Re}\left[\frac{r_{s}\left(r_{p}+r_{s}\right)}{r_{s}^{2}+{\left(\frac{r_{p}+r_{s}}{k_{0}w}cot\theta_{I}\right)}^{2}+{\left(\frac{{\dot{r}}_{s}}{k_{0}w}\right)}^{2}}\right], \end{array}$$

where subscripts *s* and *p* indicate the *s* and *p* polarizations, subscripts *H* and *V* denote the horizontal and vertical incident polarizations, *λ* is the wavelength, *θ*_*I*_ is the incident angle, and ${\dot{r}}_{p,s}$ is the derivative of *r*_*p*,*s*_ with respect to *θ*_*I*_. Equation (3) can be simplified to [51].

(4) $$\begin{array}{c} \delta_{H}^{\pm }/\lambda =\mp \frac{\cot\theta_{I}}{2\pi }\text{Re}\left[1+\frac{r_{s}}{r_{p}}\right],\\ \delta_{V}^{\pm }/\lambda =\mp \frac{\cot\theta_{I}}{2\pi }\text{Re}\left[1+\frac{r_{p}}{r_{s}}\right], \end{array}$$

if $k_{0}^{2}w^{2}\gg \cot^{2}\theta_{I}$ is satisfied.

We define the spin Hall shift of the beam reflected at medium 1 as $\tilde{\delta }$ . Hereafter, the spin Hall shift refers to that of RCP beam. The incident polarization is horizontal, and air is considered as the background medium. For the calculations, we use the following parameters: Δ*W* = 4 µm and *ϵ* = 0.01. The laser diameter is given as 0.7 mm, and the focal lengths of the lenses before and after reflection are set to 30 and 200 mm, respectively. The calculations in this section are performed using a home-built code and cross-checked using a rigorous coupled-wave analysis-based software, MAXIM [52]. The reflection coefficients at a single interface and multiple interfaces are calculated using Fresnel equations and the transfer-matrix method [53], respectively.

Because the reflection coefficients are directly linked to the index, $\tilde{\delta }$ varies with changes in *n* (Figure 2b). According to Eq. (2), small $\tilde{\delta }$ that changes drastically in response to *n*, i.e., small $\tilde{\delta }$ and large $\frac{d\tilde{\delta }}{dn}\;$ , is favorable for achieving high precision. However, $\tilde{\delta }$ and its derivative are determined by *n*, which may result in precision that is not sufficiently high. For instance, $\tilde{\delta }/\lambda$ varies drastically only near the Brewster angle. However, $\tilde{\delta }/\lambda$ near this regime is of the order of 1 (Figure 2b), which is not sufficiently weak to satisfy the weak-coupling condition (Eq. (1)) for a small *α*. Consequently, the index resolution $\Delta \tilde{n}$ obtained using Eq. (2) is not sufficiently small (Figure 2c and d). Whereas $\Delta \tilde{n}$ reaches zero at the Brewster angle (Figure 2c, inset), unfortunately, $\tilde{\delta }$ diverges as *θ*_*I*_ deviates from the Brewster angle and hence, the weak measurement is unreliable (Figure 2e) due to the violation of Eq. (1). To examine the validity of the weak measurement in this regime, *W* is calculated by multiplying the spatial profile of the reflected beam by the Jones matrix of the postselection. Indeed, the discrepancy between the spin Hall shift obtained by dividing the numerically simulated *W* by *A* (Figure 2e, blue markers) and $\tilde{\delta }$ calculated by Eq. (3) (black curve) confirms the unreliability of the measurement. Except this regime, $\tilde{\delta }$ has a subwavelength but finite values, and $\Delta \tilde{n}$ is of the order of 10^−2^ or greater in the entire parameter space in our simulation (see Methods for details).

We now imagine a subwavelength-thick slab (*t* < *λ*) with an index of *n*_*s*_ spaced by *d* < *λ* above medium 1 (Figure 2a, gray slab). The lateral dimension of the slab is irrelevant to the results as long as it covers the entire incidence. In the presence of the slab, the beams that are multiple-reflected inside the slab and the air spacer are spatially overlapped. Because of this spatial overlap, we treat the multiple-reflected beam as a single-reflected beam, instead of considering light evolution during the multiple reflections and the resultant SHEL as in [54, 55]. It exhibits a distinct spin Hall shift, defined by *δ*, which generally differs from $\tilde{\delta }$ but contains all the information of the indices and thickness of the media, such as *n*_*s*_, *t*, *d*, and *n*. It implies that *n* can be derived from *δ* if all other parameters are given. Importantly, the proper use of the slab can significantly enhance precision by making the shift cross zero. For numerical verification, we set *θ*_*I*_ = 25°, *λ* = 633 nm, *t* = 0.2*λ*, and *n*_*s*_ = 0.5 and calculate *δ*/*λ* while scanning *n* and *d* (Figure 2f). Two curves along which *δ*/*λ* = 0 are observed at *n* > 1.15. It indicates that along these curves, Δ*n* can be infinitesimal according to Eq. (2). It is confirmed by Figure 2g, which shows Δ*n* → 0 along the same curves.

For better understanding, we examine *δ* and Δ*n* for a fixed *d* (Figure 2h and i). Notably, *δ*/*λ* = 0 shown in Figure 2h is clearly distinct from $\tilde{\delta }/\lambda =0$ (Figure 2b) considering that the spin Hall shift changes gradually across zero as *n* changes (compare Figure 2h with Figure 2d). Therefore, whereas the weak measurement is invalid when $\tilde{\delta }$ approaches the Brewster angle (Figure 2d), the spin Hall shift obtained by the simulated *W*/*A* agrees well with *δ* near *δ* = 0 (Figure 2h). The discrepancy between the two spin Hall shifts near *n* = 1 and *n* = 1.3 (gray lines) originates from the large *δ* that induces the breakdown of Eq. (1). Other parameters, such as *α*, *W*, and *A*, can be found in Supplementary Information, Section S3.

Interestingly, *δ* = 0 allows *α* to be arbitrarily small according to Eq. (1), resulting in an arbitrarily large *A* and, consequently, an infinitesimal Δ*n*. Indeed, it makes Δ*n* converge to zero without degrading the reliability of the regime (Figure 2i, green line and inset) and opens a route toward the ultrahigh-precision measurement. Additionally, the monotonic change in *δ* near *δ* = 0 enables the one-to-one identification of *n* by measuring *δ*. In contrast, when *δ* reaches a local maximum or minimum, the resolution deteriorates (Δ*n* → ∞) because $\frac{d\delta }{dn}=0$ (Figure 2h and i, gray lines). Note that when *δ* exceeds *w*, Eq. (2) becomes inaccurate because its assumption (*α* < 45°) fails.

**Figure 3:**
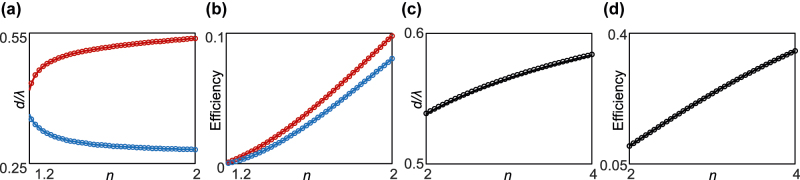
The precision enhancement for the broad *n* range. (a) *d*/*λ* for Δ*n* → 0 and (b) efficiency along the curves in (a) for 1.15 ≤ *n* ≤ 2. (c) *d*/*λ* for Δ*n* → 0 and (d) efficiency along the curve in (c) for the higher index regime (2 ≤ *n* ≤ 4).

The index range that can be detected with high precision can be tuned by adjusting *d* (see Figure 3). Two curves exist, along which Δ*n* → 0 at 1.15 ≤ *n* ≤ 2 (Figures 2f and 3a). Therefore, by controlling the spacing between the target interface and the index-below-unity slab, a broad range of indices can be measured with high precision using weak measurements. The relation between *n* and *d* (Figure 3a) implies that the index, which we aim to measure, should be known prior to the measurement. However, considering that the high-precision measurements are generally conducted in a narrow specified regime, this issue does not impose a constraint on the practicality. The efficiencies, which are equal to the intensities of the reflected beam, along the curves are below 10% (Figure 3b) but still are sufficient for the measurement, especially compared with those near the Brewster angle. The measurable index range can be further broadened, for example, to cover 2 ≤ *n* ≤ 4, by changing *d* (Figure 3c and d). It proves the versatility of nanophotonic-assisted precision enhancement for sensing applications.

Although we assume that the medium above the index-below-unity slab is air for the sake of simplicity, our concept remains valid when the superstrate has an index >1 by changing *θ*_*I*_ (see Supplementary Information, Section S4). Therefore, aside from technical difficulties, this method is experimentally feasible by positioning an index-below-unity film coated on a dielectric substrate upside-down. Furthermore, precision enhancement remains effective even when the index-below-unity slab is lossy (see Supplementary Information, Section S5). Considering that the reflection coefficients are determined by the relative ratio between the indices instead of their absolute values, the air spacing can be replaced by a medium with a higher index, such as in oil immersion, if necessary.

### 2.3 Precision enhancement using a metasurface

**Figure 4:**
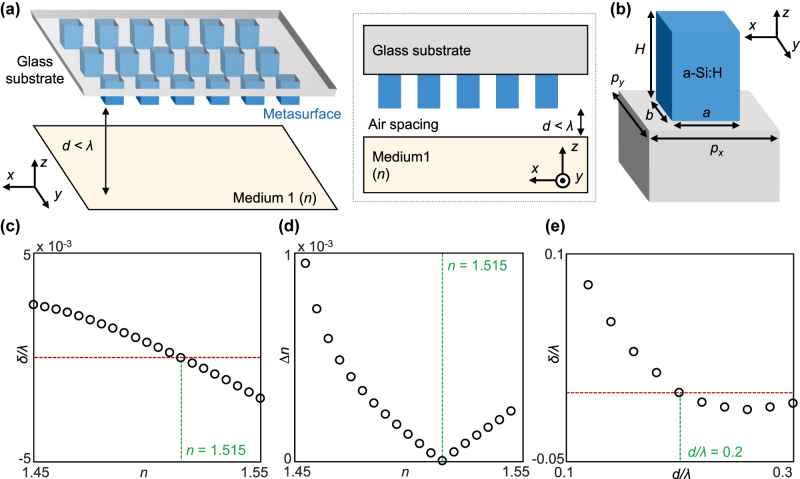
Precision enhancement using a metasurface. (a) Schematic of metasurface placed near target interface. Inset: the same schematic in the *z*–*x* plane. (b) Unit cell of the metasurface. Geometric parameters are given as: *a* = 268, *b* = 354, *H* = 371, *p*_*x*_ = 341, and *p*_*y*_ = 443 nm. The rod is made of hydrogenated amorphous silicon (a-Si: H). The incident plane is the *z*–*x* plane. (c) Spin Hall shift and (d) corresponding index resolution for various values of *n* when *d* = 0.2*λ*. (e) Spin Hall shift for various *d* values when *n* = 1.515. The green lines represent *δ* = 0. In all cases, *θ*_*I*_ = 25°.

Naturally occurring or artificially engineered materials that have indices below unity are limited; some examples include metals in the ultraviolet, randomly dispersed metallic nanoparticles in a dielectric host in the visible, and inorganic compounds in the mid-infrared regime. Scarcity of such materials and high optical losses of them make an experimental demonstration of the concept introduced in Section 2.2 challenging. Therefore, a pragmatic nanophotonic system that does not include exotic bulk properties is in demand. In this section, a metasurface-enabled approach for precision enhancement is introduced as an alternative (Figure 4a). The basic principle of the precision enhancement is the same as in Section 2.2; positioning the metasurface in the vicinity of the target interface alters the light–matter interactions at the subwavelength scale and results in a modified SHEL. The role of the metasurface is to adjust the relative amplitude and phase difference of two reflection coefficients (*r*_*s*_ and *r*_*p*_) to make the spin Hall shift cross zero.

A metasurface consists of periodically arranged rectangular rods made of hydrogenated amorphous silicon (Figure 4b). The simulations in this section are conducted using commercial software based on the finite-element-method (COMSOL Multiphysics). The refractive indices of the glass and a-Si:H are 1.457 and 3.503 + 0.046*i*, respectively. Five parameters (height, rod length, rod width, and two periodicities) are optimized to minimize |Re(1 + *r*_*s*_/*r*_*p*_)| for *n* = 1.515, *d* = 0.2*λ*, and *θ*_*I*_ = 25° to achieve a zero spin Hall shift under horizontally polarized incidence (Eq. (4)). Here, *r*_*s*_ and *r*_*p*_ are the Fresnel coefficients of the entire system comprising the metasurface on the glass substrate, air spacing, and medium 1. The electric field distribution near the metasurface can be found in Supplementary Information, Section S6. The spin Hall shift of the entire composite system shown in Figure 4a crosses *δ* = 0 smoothly at *n* = 1.515 (Figure 4c, green line). Consequently, Δ*n* is of the order of 10^−4^ at 1.45 ≤ *n* ≤ 1.55 and, in particular, converges to zero at *n* = 1.515 (Figure 4d). High Δ*n* near *n* = 1.45 is attributed to small $\frac{d\delta }{dn}.\;$ In addition, *δ* varies gradually as *d* changes, indicating that the measurable index range can be adjusted by controlling *d* (Figure 4e). These results demonstrate that a single-layer all-dielectric metasurface with a subwavelength thickness can be implemented in a weak measurement and can significantly enhance its precision.

### 2.4 Discussion

For completeness, we discuss experimental possibilities, limitations, and further details of our method. While our work is purely theoretical, the scheme is experimentally feasible, especially when combined with precise positioning techniques, which are being used in near-field scanning and near-field heat transfer communities. To avoid technical challenges, coating the target interface with a thin film can be an alternative to make *δ* = 0 without requiring the precise positioning at the expanse of nondestructiveness; however, this method impairs the sample and is not considered in our study.

One limitation of this method is that the properties of the index-below-unity slab or the metasurface such as the slab thickness and nanorod dimensions are dependent on the target interface. Therefore, the examination of a different interface requires optimization and fabrication of a new slab or metasurface. Our method is more suitable when rough information of the target interface is given and fixed and the extremely precise inspection is required, such as in medical diagnosis or to observe small perturbations, rather than for investigation of a completely unknown interfaces. As stated earlier, this issue is not a serious limitation, given that the high-precision measurements are usually performed in a specific range.

If the slab or metasurface is imperfect, i.e., if the slab has a nonzero surface roughness or the geometric dimensions of the metasurface deviate slightly from the optimized values, *δ* may also deviate from its desired value (which is zero) and can restrict the precision in reality. Finally, note that changes in the constant parameters in our calculations (Δ*W*, *ϵ*, focal lengths of two lenses, etc.) alter the spin Hall shift [10, 46] and resolution of two instances (with and without the slab or the metasurface) together, and our precision enhancement is valid under other parameters.

## 3 Conclusions

In conclusion, by leveraging the index-below-unity slab and metasurface, we present two nanophotonic-assisted approaches to identify unknown parameters of interfaces with high precision or to detect infinitesimal changes using a weak measurement method. Whereas previous studies have controlled setup parameters such as the focal lengths and postselection angles for better precision, here we directly control the SHEL by placing the subwavelength-thick slab and/or metasurface near the target interface and prove that precision can be infinite, in principle, by making the spin Hall shift cross zero. Our methods provide an extremely high-precision measurement of the refractive index without any specified model and can be straightforwardly extended to the detection of other unknown quantities, such as geometric parameters, electric/magnetic properties, and concentration. We believe that our work serves as the first attempt to implement nanophotonics to enhance the SHEL-based precision engineering and will be implemented in practical applications of ultrahigh-precision measurements such as diagnosis, monitoring, and chemical sensing.

## Supplementary Material

Supplementary Material Details
