# Supplementary material for: Nanophotonic-assisted precision enhancement of weak measurement using spin Hall effect of light
Source: Nanophotonics. 2022 Sep 20;11(20):4591–600. doi: 10.1515/nanoph-2022-0447 (PMC11501708; doi:10.1515/nanoph-2022-0447)
Supplement: Supplementary file 1 — Supplementary Material Details [file j_nanoph-2022-0447_suppl_001.pdf]

Supporting Information of "Nanophotonic-assisted precision enhancement of weak measurement using spin Hall effect of light"

Minkyung Kim[1,2], Dasol Lee[3], Yeseul Kim[1], and Junsuk Rho[1,4,5*]

[1]Department of Mechanical Engineering, Pohang University of Science and Technology (POSTECH), Pohang 37673, Republic of Korea
[2]School of Mechanical Engineering, Gwangju Institute of Science and Technology (GIST), Gwangju 61005, Republic of Korea
[3]Department of Biomedical Engineering, Yonsei University, Wonju 26493, Republic of Korea
[4]Department of Chemical Engineering, Pohang University of Science and Technology (POSTECH), Pohang 37673, Republic of Korea
[5]POSCO-POSTECH-RIST Convergence Research Center for Flat Optics and Metaphotonics, Pohang 37673, Republic of Korea
*jsrho@postech.ac.kr

S1 Derivation of amplification factor

This section describes the detailed theory of the amplification principle and derivation of the amplification factor $A = F\cot\alpha$ using a purely optical approach. Under horizontally polarized incidence, the beam that is reflected at an interface with Fresnel reflection coefficients r_s and r_p can be expressed as

$$\psi(x, y, z_1) \propto \begin{pmatrix} r_p - i\frac{x}{z_R+iz_1}\dot{r}_p \\ i\frac{y\cot\theta_i}{z_R+iz_1}(r_p + r_s) - \frac{xy\cot\theta_i}{(z_R+iz_1)^2}(\dot{r}_p + \dot{r}_s) \end{pmatrix} \exp\Big(-\frac{k_0}{2}\frac{x^2+y^2}{z_R+iz_1}\Big), \tag{S1}$$

where z_1 is the propagation distance since the reflection and (x, y) is defined at the surface normal to the propagation. The beam after passing through the second lens with the focal length of f_2 can be obtained by replacing z_R with $\tilde{z}_R = (1 + F^2)z_R$, where $F = z_1/z_R$, and z_1 with z_2, where z_2 is the propagation distance since the second lens:

$$\psi_{\text{after lens}}(x, y, z_2) \propto \begin{pmatrix} r_p - i\frac{x}{z_R+iz_1}\dot{r}_p \\ i\frac{y\cot\theta_i}{z_R+iz_1}(r_p + r_s) - \frac{xy\cot\theta_i}{(z_R+iz_1)^2}(\dot{r}_p + \dot{r}_s) \end{pmatrix} \exp\Big(-\frac{k_0}{2}\frac{x^2+y^2}{\tilde{z}_R+iz_2}\Big). \tag{S2}$$

The beam profile after the postselection can be obtained by multiplying its Jones vector $(-\sin\alpha, \cos\alpha)$ as

$$\psi_{\text{after postselection}}(x=0, y, z_2) \propto \exp\Big(-\frac{k_0}{2}\frac{y^2}{\tilde{z}_R+iz_2} - i\frac{y\cot\alpha}{z_R+iz_1}(1+\frac{r_s}{r_p})\cot\theta_i\Big), \tag{S3}$$

by using the first-order Taylor expansion, $\exp\triangle \approx 1 + \triangle$ for sufficiently small $\triangle$. Note that this assumption is valid under the weak regime (Eq. 1 in the main manuscript). From Eq. S3, the amplitude of the postselected beam can be obtained as

$$|\psi| \propto \exp\Big(-\frac{k_0}{2}\frac{\tilde{z}_R}{\tilde{z}_R^2+z_2^2}\Big[y + \frac{z_1}{z_R^2+z_1^2}\frac{\tilde{z}_R^2+z_2^2}{\tilde{z}_R}\text{Re}(1+\frac{r_s}{r_p})\frac{\cot\theta_i}{k_0}\cot\alpha\Big]^2\Big). \tag{S4}$$

This equation demonstrates that the postselected beam is displaced from $y = 0$ by

$$\langle y \rangle = \frac{z_1}{z_R^2 + z_1^2} \frac{\tilde{z}_R^2 + z_2^2}{\tilde{z}_R} \delta \cot \alpha, \tag{S5}$$

where $\delta = -\mathrm{Re}(1 + \frac{r_s}{r_p}) \frac{\cot \theta_i}{k_0}$ is the well-known spin Hall shift formula. Provided that the measurement parameters such as the focal lengths are determined to produce large $F \gg 1$ for the amplification, Eq. S5 can be approximated to $\langle y \rangle = \delta F \cot \alpha$, which clearly shows the amplification factor formula, that is, $A = F \cot \alpha$.

S2 Derivation of Equation 2

Equation 2 in the main manuscript can be derived as following:

$$\begin{aligned} \Delta n &= \left| \frac{dn}{dW} \Delta W \right| \\ &= \left| \frac{dn}{d\delta} \frac{\delta}{W} \Delta W \right| \\ &= \left| \left(\frac{d\delta}{dn} \right)^{-1} \frac{\Delta W}{F \cot \alpha} \right| \\ &= \left| \delta \left(\frac{d\delta}{dn} \right)^{-1} \frac{\Delta W}{\epsilon F w} \right|, \end{aligned} \tag{S6}$$

where $\delta = \epsilon w \tan \alpha$ is used for the last equality. Note that $\delta = \epsilon w \tan \alpha$ is the alternate expression of

$$\frac{|\delta|}{w} \ll \tan \alpha, \tag{S7}$$

which is equal to Equation 1 in the main manuscript for small α ($< 45°$).

S3 Postselection angle, weak value, and amplification factor

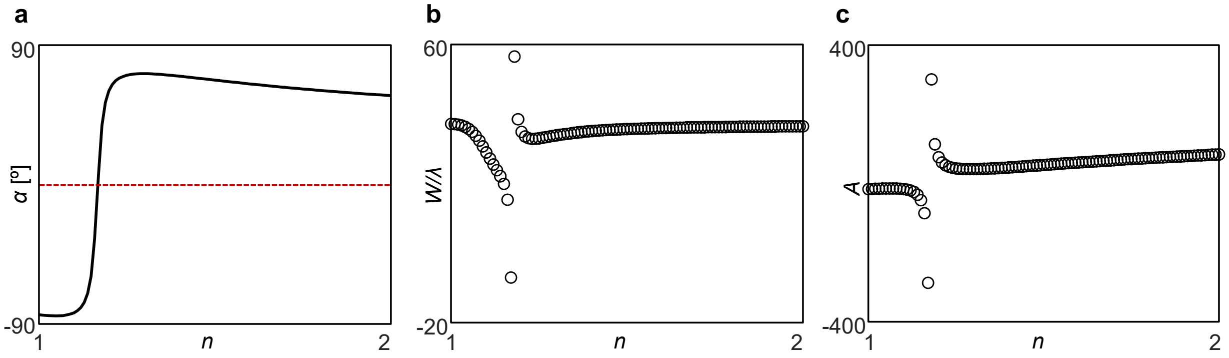

Figure S1. Supplementary data for Fig. 2h in the main manuscript. (a) α, (b) W normalized by λ, and (c) A.

Fig. S4 shows supplementary data for Fig. 2h in the main manuscript. The spin Hall shift that reaches zero at $n = 1.515$ allows α to be arbitrarily small, which amplifies the W significantly. Because we enforce α to be dependent on the spin Hall shift, α in this regime is near $-90°$, which indicates no postselection at all.

S4 Precision enhancement using the index-below-unity slab on a dielectric substrate

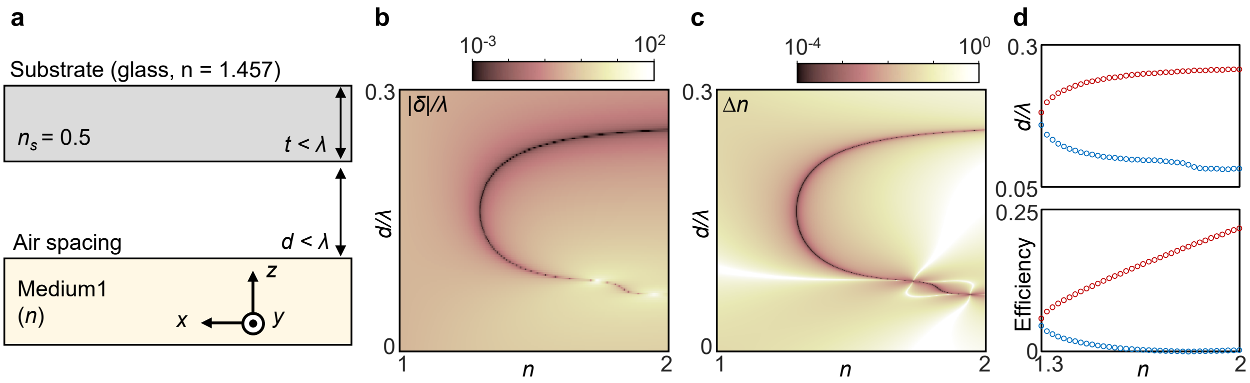

Figure S2. Precision enhancement using the index-below-unity slab on a glass substrate (n = 1.457). (a) Schematic and (b) the magnitude of the spin Hall shift and (c) the corresponding index resolution. (d) d/λ for $\Delta n \to 0$ and the efficiencies along the same curves.

In the main manuscript, the background medium is considered as air, which makes the index-below-unity slab levitate in the air. However, our method works well when the medium above the slab has index beyond unity. Fig. S2 presents the precision enhancement using the index-below-unity slab on a dielectric substrate. Along the similar curves, $\delta = 0$ and therefore $\Delta n \to 0$ are observed. In particular, the red curve shown in Fig. S2d will be a good candidate along which the index can be measured with high precision and moderate efficiency. Note that in experiments, the SHEL at the opposite side of the substrate will be also included, but can be excluded in the data [1].

S5 Precision enhancement using a lossy index-below-unity slab

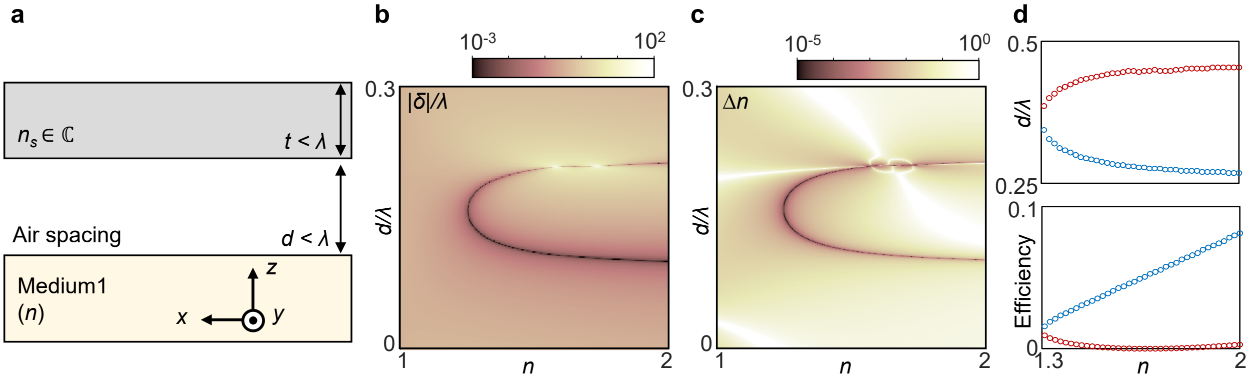

Figure S3. Precision enhancement using a lossy index-below-unity slab. (a) Schematic and (b) the magnitude of the spin Hall shift and (c) the corresponding index resolution. (d) d/λ for $\Delta n \to 0$ and the efficiencies along the same curves.

Because of the dispersive nature of index-below-unity or index-near-zero materials, the slab

may have nonzero optical losses. Fig. S3 demonstrates that our scheme also works when the index-below-unity slab is lossy. The blue curve shown in Fig. S3 can be used to achieve high precision and efficiency.

S6 Field profiles near the metasurface

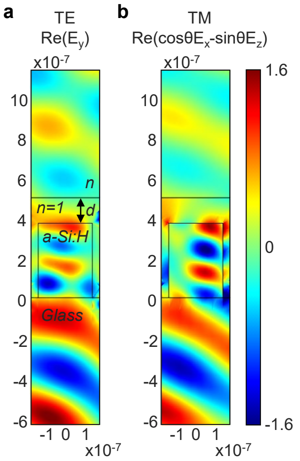

Figure S4. Electric field profiles near the metasurface under (a) transverse electric and (b) transverse magnetic incidences. The field magnitude is normalized by the amplitude of the incidence. Incident angle is 25° as in the main manuscript.

For completeness, electric field profiles near the metasurface under transverse electric (TE) and tranverse magnetic (TM) modes are presented in Fig. S4. Whereas the reflection coefficients of the TE and TM modes are generally different under oblique incidence at most interfaces and thus producing nonzero spin Hall shift, the reflection coefficients at this artificially engineered interface support $|\mathrm{Re}(1 + r_s/r_p)| \approx 0$, leading to the negligible spin Hall shift.

References

[1] O. Hosten and P. Kwiat. Observation of the spin Hall effect of light via weak measurements. *Science* **319**(5864), 787–790 (2008).
